# Supplementary material for: Computational Modelling of Tunicamycin C Interaction with Potential Protein Targets: Perspectives from Inverse Docking with Molecular Dynamic Simulation
Source: Curr Issues Mol Biol. 2025 May 8;47(5):339. doi: 10.3390/cimb47050339 (PMC12110290; doi:10.3390/cimb47050339)
Supplement: Supplementary file 1 [file cimb-47-00339-s001.zip › cimb-3479662-supplementary.pdf]

Table S.1. Docking results of Tunicamycin with protein targets

PDB ID	Protein	Docking Score
2h26	T-cell surface glycoprotein CD1b (CD1B_HUMAN)	11
2r59	Leukotriene A-4 hydrolase (LKHA4_HUMAN)	10.6
2vqq	Histone deacetylase 4 (HDAC4_HUMAN)	10.2
2gvj	Nicotinamide phosphoribosyltransferase (NAMPT_HUMAN)	10.1
1s9i	Dual specificity mitogen-activated protein kinase kinase 2 (MP2K2_HUMAN)	9.9
2po6	T-cell surface glycoprotein CD1d (CD1D_HUMAN)	9.8
2f38	Aldo-keto reductase family 1 member C3 (AK1C3_HUMAN)	9.7
2h44	cGMP-specific 3',5'-cyclic phosphodiesterase (PDE5A_HUMAN)	9.6
2hh5	Cathepsin S (CATS_HUMAN)	9.6
2w4q	Prostaglandin reductase 2 (PTGR2_HUMAN)	9.6
1e9a	Thymidylate kinase (KTHY_HUMAN)	9.5
1ros	Macrophage metalloelastase (MMP12_HUMAN)	9.5
1ohk	Dihydrofolate reductase (DYR_HUMAN)	9.4
1d1j	Profilin-2 (PROF2_HUMAN)	9.2
1egc	Medium-chain specific acyl-CoA dehydrogenase, mitochondrial (ACADM_HUMAN)	9.2
1ezf	Squalene synthetase (FDFT_HUMAN)	9.2
1kkq	Nuclear receptor corepressor 2 (NCOR2_HUMAN)	9.2
1mc5	Alcohol dehydrogenase class-3 (ADHX_HUMAN)	9.2
1t46	Mast/stem cell growth factor receptor (KIT_HUMAN)	9.2
1w78	Bifunctional protein folC (FOLC_ECOLI)	9.2
1wuu	Galactokinase(GALK1_HUMAN)	9.2
2abj	Branched-chain-amino-acid aminotransferase, cytosolic (BCAT1_HUMAN)	9.2
2fv5	ADAM 17 (ADA17_HUMAN)	9.2
2ewy	Beta-secretase 2 (BACE2_HUMAN)	9.1
2nni	Cytochrome P450 2C8 (CP2C8_HUMAN)	9.1
2rjp	A disintegrin and metalloproteinase with thrombospondin motifs 4 (ATS4_HUMAN)	9.1
1ksw	Proto-oncogene tyrosine-protein kinase Src (SRC_HUMAN)	9
1kws	Galactosylgalactosylxylosylprotein 3-beta-glucuronosyltransferase 3	9
1sg0	Ribosyldihydronicotinamide dehydrogenase [quinone] (NQO2_HUMAN)	9
2hi4	Cytochrome P450 1A2 (CP1A2_HUMAN)	9
2w0b	Cytochrome P450 51 (CP51_MYCTU)	9
2wax	Probable ATP-dependent RNA helicase DDX6 (DDX6_HUMAN)	9
1ish	ADP-ribosyl cyclase 2 (BST1_HUMAN)	8.9
1qu3	Isoleucyl-tRNA synthetase (SYI1_STAAU)	8.9
1s9j	Dual specificity mitogen-activated protein kinase kinase 1(MP2K1_HUMAN)	8.9
2fb8	B-Raf proto-oncogene serine/threonine-protein kinase(BRAF1_HUMAN)	8.9
2ovz	Matrix metalloproteinase-9 (MMP9_HUMAN)	8.9
1h22	Acetylcholinesterase (ACES_TORCA)	8.8
1imb	Inositol monophosphatase (IMPA1_HUMAN)	8.8
1onq	T-cell surface glycoprotein CD1a (CD1A_HUMAN)	8.8
1qip	Lactoylglutathione lyase (LGUL_HUMAN)	8.8
1so2	cGMP-inhibited 3',5'-cyclic phosphodiesterase B (PDE3B_HUMAN)	8.8
2nru	Interleukin-1 receptor-associated kinase 4 (IRAK4_HUMAN)	8.8
2x6v	T-box transcription factor TBX5 (TBX5_HUMAN)	8.8

1byg	Tyrosine-protein kinase CSK(CSK_HUMAN)	8.7
1og5	Cytochrome P450 2C9 (CP2C9_HUMAN)	8.7
2c0i	Tyrosine-protein kinase HCK (HCK_HUMAN)	8.7
2e3r	Collagen type IV alpha-3-binding protein (C43BP_HUMAN)	8.7
2oc2	Angiotensin-converting enzyme (ACE_HUMAN)	8.7
2x7h	Zinc-binding alcohol dehydrogenase domain-containing protein 2 (ZADH2_HUMAN)	8.7
3ant	Epoxide hydrolase 2 (HYES_HUMAN)	8.7
1r4l	Angiotensin-converting enzyme 2 (ACE2_HUMAN)	8.6
1zdt	Steroidogenic factor 1 (STF1_HUMAN)	8.6
2bdm	Cytochrome P450 2B4 (CP2B4_RABIT)	8.6
2bil	Proto-oncogene serine/threonine-protein kinase Pim-1 (PIM1_HUMAN)	8.6
2jev	Diamine acetyltransferase 1 (SAT1_HUMAN)	8.6
2q7r	Arachidonate 5-lipoxygenase-activating protein (AL5AP_HUMAN)	8.6
2qg6	Nicotinamide riboside kinase 1(NRK1_HUMAN)	8.6
2vn9	Calcium/calmodulin-dependent protein kinase type II delta chain (KCC2D_HUMAN)	8.6
2woe	ADP-ribosyl-[dinitrogen reductase] glycohydrolase (DRAG_RHORU)	8.6
2xbj	Serine/threonine-protein kinase Chk2 (CHK2_HUMAN)	8.6
1gnj	Serum albumin (ALBU_HUMAN)	8.5
1ogu	Cyclin-A2 (CCNA2_HUMAN)	8.5
1s8c	Heme oxygenase 1 (HMOX1_HUMAN)	8.5
2z65	Lymphocyte antigen 96 Toll-like receptor 4(LY96_HUMAN/TLR4_HUMAN)	8.5
1d4l	Gag-Pol polyprotein (POL_HV1A2)	8.4
1rgy	Beta-lactamase (AMPC_CITFR)	8.4
1rne	Renin (RENI_HUMAN)	8.4
2jiv	Epidermal growth factor receptor (EGFR_HUMAN)	8.4
2r3v	Mevalonate kinase(KIME_HUMAN)	8.4
2vgq	Mitochondrial antiviral-signaling protein (MAVS_HUMAN)	8.4
2vz6	Calcium/calmodulin-dependent protein kinase type II alpha chain (KCC2A_HUMAN)	8.4
3a4o	Tyrosine-protein kinase Lyn(LYN_HUMAN)	8.4
1esv	Gelsolin (GELS_HUMAN)	8.3
1k9s	Purine nucleoside phosphorylase deoD-type (DEOD_ECOLI)	8.3
1lqv	Endothelial protein C receptor Vitamin K-dependent protein	8.3
1pk0	Calmodulin (CALM_HUMAN)	8.3
1pl6	Sorbitol dehydrogenase (DHSO_HUMAN)	8.3
1t40	Aldose reductase (ALDR_HUMAN)	8.3
2gz5	Methionine aminopeptidase 1 (AMPM1_HUMAN)	8.3
2jed	Protein kinase C theta type (KPCT_HUMAN)	8.3
2ntj	Enoyl-[acyl-carrier-protein] reductase [NADH] (INHA_MYCTU)	8.3
2qpj	Neprilysin (NEP_HUMAN)	8.3
2qt0	Nicotinamide riboside kinase 1 (NRK1_HUMAN)	8.3
1bbp	Bilin-binding protein (BBP_PIEBR)	8.2
1d8d	GTPase KRas (RASK_HUMAN)	8.2
1fm9	Peroxisome proliferator-activated receptor gamma (PPARG_HUMAN)	8.2
1hh4	Ras-related C3 botulinum toxin substrate 1 (RAC1_HUMAN)	8.2
1qzy	Methionine aminopeptidase 2 (AMPM2_HUMAN)	8.2
1s5o	Carnitine O-acetyltransferase (CACP_HUMAN)	8.2

1ysw	Apoptosis regulator Bcl-2 (BCL2_HUMAN)	8.2
2iwi	Serine/threonine-protein kinase Pim-2 (PIM2_HUMAN)	8.2
1caq	Stromelysin-1 (MMP3_HUMAN)	8.1
1i4f	HLA class I histocompatibility antigen, A-2 alpha chain Melanoma-associated antigen 4(1A02_HUMAN/MAGA4_HUMAN)	8.1
1iz2	Alpha-1-antitrypsin (A1AT_HUMAN)	8.1
1nf7	Inosine-5'-monophosphate dehydrogenase 2 (IMDH2_HUMAN)	8.1
1rc1	Trifunctional purine biosynthetic protein adenosine-3 (PUR2_HUMAN)	8.1
2cke	Death-associated protein kinase 2 (DAPK2_HUMAN)	8.1
2cmw	Casein kinase I isoform gamma-1 (KC1G1_HUMAN)	8.1
2dr6	Acriflavine resistance protein B (ACRB_ECOLI)	8.1
2hw7	MAP kinase-interacting serine/threonine-protein kinase 2(MKNK2_HUMAN)	8.1
2io6	Wee1-like protein kinase (WEE1_HUMAN)	8.1
2ouq	cAMP and cAMP-inhibited cGMP 3',5'-cyclic phosphodiesterase 10A	8.1
2v5x	Histone deacetylase 8 (HDAC8_HUMAN)	8.1
2vct	Glutathione S-transferase A2 (GSTA2_HUMAN)	8.1
2vx3	Dual specificity tyrosine-phosphorylation-regulated kinase 1A (DYR1A_HUMAN)	8.1
2z8i	Gamma-glutamyltranspeptidase (GGT_ECOLI)	8.1
1tu6	Cathepsin K (CATK_HUMAN)	8
2jih	A disintegrin and metalloproteinase with thrombospondin motifs 1 (ATS1_HUMAN)	8
2q32	Heme oxygenase 2 (HMOX2_HUMAN)	8
2vc2	Integrin alpha-IIb Integrin beta-3(ITA2B_HUMAN/ITB3_HUMAN)	8
2xef	Glutamate carboxypeptidase 2 (FOLH1_HUMAN)	8
1dmw	Phenylalanine-4-hydroxylase (PH4H_HUMAN)	7.9
1s0x	Nuclear receptor ROR-alpha (RORA_HUMAN)	7.9
1zrz	Protein kinase C iota type (KPCI_HUMAN)	7.9
2.00E+82	D-amino-acid oxidase (OXDA_HUMAN)	7.9
2ojj	Mitogen-activated protein kinase 1 (MK01_HUMAN)	7.9
2qrn	Deoxycytidine kinase(DCK_HUMAN)	7.9
2v3e	Glucosylceramidase (GLCM_HUMAN)	7.9
2vle	Aldehyde dehydrogenase, mitochondrial (ALDH2_HUMAN)	7.9
1fco	Beta-lactamase (AMPC_ECOLI)	7.8
1ib1	14-3-3 protein zeta/delta (1433Z_HUMAN)	7.8
1nue	Nucleoside diphosphate kinase B(NDKB_HUMAN)	7.8
1pmn	Mitogen-activated protein kinase 10 (MK10_HUMAN)	7.8
1skx	Nuclear receptor subfamily 1 group I member 2 (NR1I2_HUMAN)	7.8
1tuf	Diaminopimelate decarboxylase (DCDA_METJA)	7.8
1z11	Cytochrome P450 2A6 (CP2A6_HUMAN)	7.8
2bu7	[Pyruvate dehydrogenase [lipoamide]] kinase isozyme 2, mitochondrial (PDK2_HUMAN)	7.8
2clq	Mitogen-activated protein kinase kinase kinase 5(M3K5_HUMAN)	7.8
2eal	Galectin-9 (LEG9_HUMAN)	7.8
2euf	Cell division protein kinase 6 (CDK6_HUMAN)	7.8
2v4m	Glucosamine--fructose-6-phosphate aminotransferase [isomerizing] 1	7.8
2vag	Dual specificity protein kinase CLK1 (CLK1_HUMAN)	7.8
2vd5	Myotonin-protein kinase (DMPK_HUMAN)	7.8
2xb7	ALK tyrosine kinase receptor (ALK_HUMAN)	7.8

3acl	Pirin (PIR_HUMAN)	7.8
1a5h	Tissue-type plasminogen activator (TPA_HUMAN)	7.7
1dgh	Catalase (CATA_HUMAN)	7.7
1k4t	DNA topoisomerase 1 (TOP1_HUMAN)	7.7
1nd5	Prostatic acid phosphatase (PPAP_HUMAN)	7.7
1p4r	Bifunctional purine biosynthesis protein PURH (PUR9_HUMAN)	7.7
2fs8	Tryptase beta-2 (TRYB2_HUMAN)	7.7
2h6i	Protein farnesyltransferase/geranylgeranyltransferase type-1 subunit alpha (FNTA_HUMAN)	7.7
2jam	Calcium/calmodulin-dependent protein kinase type 1G (KCC1G_HUMAN)	7.7
2nyr	NAD-dependent deacetylase sirtuin-5 (SIRT5_HUMAN)	7.7
2obd	Cholesteryl ester transfer protein (CETP_HUMAN)	7.7
2x7o	TGF-beta receptor type-1 (TGFR1_HUMAN)	7.7
2y7j	Phosphorylase b kinase gamma catalytic chain, testis/liver isoform	7.7
3agm	cAMP-dependent protein kinase catalytic subunit alpha (KAPCA_HUMAN)	7.7
1a2n	UDP-N-acetylglucosamine 1-carboxyvinyltransferase (MURA_ECOLI)	7.6
1gnp	GTPase HRas (RASH_HUMAN)	7.6
1rv1	E3 ubiquitin-protein ligase Mdm2 (MDM2_HUMAN)	7.6
1ucn	Nucleoside diphosphate kinase A(NDKA_HUMAN)	7.6
1unl	Cyclin-dependent kinase 5 activator 1 Cell division protein kinase 5	7.6
2g0e	HTH-type transcriptional regulator qacR (QACR_STAHA)	7.6
2gl6	Creatine kinase, sarcomeric mitochondrial(KCRS_HUMAN)	7.6
2hy8	Serine/threonine-protein kinase PAK 1 (PAK1_HUMAN)	7.6
2qmj	Maltase-glucoamylase, intestinal (MGA_HUMAN)	7.6
2uym	Kinesin-like protein KIF11 (KIF11_HUMAN)	7.6
1byd	Beta-amylase (AMYB_SOYBN)	7.5
1qvn	Interleukin-2 (IL2_HUMAN)	7.5
1u59	Tyrosine-protein kinase ZAP-70(ZAP70_HUMAN)	7.5
1xdd	Integrin alpha-L (ITAL_HUMAN)	7.5
1zsl	Coagulation factor XI (FA11_HUMAN)	7.5
2chl	Casein kinase I isoform gamma-3 (KC1G3_HUMAN)	7.5
2dq7	Proto-oncogene tyrosine-protein kinase Fyn(FYN_HUMAN)	7.5
2i6a	Adenosine kinase (ADK_HUMAN)	7.5
2px6	Fatty acid synthase (FAS_HUMAN)	7.5
2q5g	Peroxisome proliferator-activated receptor delta (PPARD_HUMAN)	7.5
2shp	Tyrosine-protein phosphatase non-receptor type 11 (PTN11_HUMAN)	7.5
2v7o	Calcium/calmodulin-dependent protein kinase type II gamma chain	7.5
2vwu	Ephrin type-B receptor 4 (EPHB4_HUMAN)	7.5
2zv2	Calcium/calmodulin-dependent protein kinase kinase 2 (KKCC2_HUMAN)	7.5
1csb	Cathepsin B (CATB_HUMAN)	7.4
1doa	Cell division control protein 42 homolog (CDC42_HUMAN)	7.4
1ek6	UDP-glucose 4-epimerase (GALE_HUMAN)	7.4
1h27	Cyclin-A2@Cyclin-dependent kinase inhibitor	7.4
1hmr	Fatty acid-binding protein, heart (FABPH_HUMAN)	7.4
1j96	Aldo-keto reductase family 1 member C2 (AK1C2_HUMAN)	7.4
1kfy	Fumarate reductase flavoprotein subunit (FRDA_ECOLI)	7.4
1nde	Estrogen receptor beta (ESR2_HUMAN)	7.4
1p7c	Thymidine kinase (KITH_HHV11)	7.4

1q5h	Deoxyuridine 5'-triphosphate nucleotidohydrolase, mitochondrial (DUT_HUMAN)	7.4
1rm8	Matrix metalloproteinase-16 (MMP16_HUMAN)	7.4
1y6q	5'-methylthioadenosine/S-adenosylhomocysteine nucleosidase (MTNN_ECOLI)	7.4
1yfh	Methylated-DNA--protein-cysteine methyltransferase (MGMT_HUMAN)	7.4
2ayr	Estrogen receptor (ESR1_HUMAN)	7.4
2buj	Serine/threonine-protein kinase 16(STK16_HUMAN)	7.4
2bxr	Amine oxidase [flavin-containing] A (AOFA_HUMAN)	7.4
2f57	Serine/threonine-protein kinase PAK 7 (PAK7_HUMAN)	7.4
2q80	Geranylgeranyl pyrophosphate synthetase (GGPPS_HUMAN)	7.4
2w4o	Calcium/calmodulin-dependent protein kinase type IV (KCC4_HUMAN)	7.4
3a7h	Serine/threonine-protein kinase 24(STK24_HUMAN)	7.4
1b86	Hemoglobin subunit alpha beta(HBA_HUMAN/HBB_HUMAN)	7.3
1cm8	Mitogen-activated protein kinase 12(MK12_HUMAN)	7.3
1i7b	S-adenosylmethionine decarboxylase proenzyme (DCAM_HUMAN)	7.3
1lpg	Coagulation factor X (FA10_HUMAN)	7.3
1mrl	Streptogramin A acetyltransferase (VATD_ENTFC)	7.3
1nny	Tyrosine-protein phosphatase non-receptor type 1 (PTN1_HUMAN)	7.3
1qhy	Chloramphenicol 3-O phosphotransferase (CPT_STRVL)	7.3
1xd0	Pancreatic alpha-amylase (AMYP_HUMAN)	7.3
2byi	Heat shock protein HSP 90-alpha (HS90A_HUMAN)	7.3
2eva	Mitogen-activated protein kinase kinase kinase 7(M3K7_HUMAN)	7.3
2fgi	Basic fibroblast growth factor receptor 1 (FGFR1_HUMAN)	7.3
2gv7	Suppressor of tumorigenicity protein 14 (ST14_HUMAN)	7.3
2hz6	Serine/threonine-protein kinase/endoribonuclease IRE1(ERN1_HUMAN)	7.3
2oat	Ornithine aminotransferase, mitochondrial (OAT_HUMAN)	7.3
2p4j	Beta-secretase 1 (BACE1_HUMAN)	7.3
2q6b	3-hydroxy-3-methylglutaryl-coenzyme A reductase (HMDH_HUMAN)	7.3
2qu6	Vascular endothelial growth factor receptor 2 (VGFR2_HUMAN)	7.3
2vdy	Corticosteroid-binding globulin (CBG_HUMAN)	7.3
2xk4	Serine/threonine-protein kinase Nek2 (NEK2_HUMAN)	7.3
3a60	Ribosomal protein S6 kinase beta-1(KS6B1_HUMAN)	7.3
1hak	Annexin A5 (ANXA5_HUMAN)	7.2
1itu	Dipeptidase 1 (DPEP1_HUMAN)	7.2
1x89	Neutrophil gelatinase-associated lipocalin (NGAL_HUMAN)	7.2
2ivu	Proto-oncogene tyrosine-protein kinase receptor ret (RET_HUMAN)	7.2
2p4i	Angiopoietin-1 receptor (TIE2_HUMAN)	7.2
2x4z	Serine/threonine-protein kinase PAK 4 (PAK4_HUMAN)	7.2
2zoq	Mitogen-activated protein kinase 3(MK03_HUMAN)	7.2
1c4y	Prothrombin (THRB_HUMAN)	7.1
1kgi	Transthyretin (TTHY_RAT)	7.1
1li4	Adenosylhomocysteinase (SAHH_HUMAN)	7.1
1rwx	Caspase-1 (CASP1_HUMAN)	7.1
2afu	Glutaminyl-peptide cyclotransferase (QPCT_HUMAN)	7.1
2az5	Tumor necrosis factor (TNFA_HUMAN)	7.1
2wu7	Dual specificity protein kinase CLK3 (CLK3_HUMAN)	7.1
1a4q	Neuraminidase (NRAM_INBBE)	7
1aj2	Dihydropteroate synthase (DHPS_ECOLI)	7

1b0f	Leukocyte elastase (ELNE_HUMAN)	7
1ec1	Gag-Pol polyprotein (POL_HV1B1)	7
1h9u	Retinoic acid receptor RXR-beta (RXRB_HUMAN)	7
1jls	Uracil phosphoribosyltransferase (UPP_TOXGO)	7
1lpa	Pancreatic triacylglycerol lipase (LIPP_HUMAN)	7
1nun	Fibroblast growth factor 10 (FGF10_HUMAN)	7
1vj9	Urokinase-type plasminogen activator (UROK_HUMAN)	7
2ar9	Caspase-9 (CASP9_HUMAN)	7
2gi7	Platelet glycoprotein VI (GPVI_HUMAN)	7
2jc6	Calcium/calmodulin-dependent protein kinase type 1D (KCC1D_HUMAN)	7
2x7k	Peptidyl-prolyl cis-trans isomerase-like 1 (PPIL1_HUMAN)	7
2zdx	[Pyruvate dehydrogenase [lipoamide]] kinase isozyme 4, mitochondrial	7
3a7e	Catechol O-methyltransferase (COMT_HUMAN)	7
1rhu	Caspase-3 (CASP3_HUMAN)	6.9
1w7n	Kynurenine--oxoglutarate transaminase 1 (KAT1_HUMAN)	6.9
2e9p	Serine/threonine-protein kinase Chk1 (CHK1_HUMAN)	6.9
2i6l	Mitogen-activated protein kinase 6(MK06_HUMAN)	6.9
2oyc	Pyridoxal phosphate phosphatase (PLPP_HUMAN)	6.9
2qnj	MAP/microtubule affinity-regulating kinase 3(MARK3_HUMAN)	6.9
1fdq	Fatty acid-binding protein, brain (FABP7_HUMAN)	6.8
1ghm	Beta-lactamase (BLAC_STAAU)	6.8
1wkw	Eukaryotic translation initiation factor 4E-binding protein 1 (4EBP1_HUMAN)	6.8
1yb1	Estradiol 17-beta-dehydrogenase 11 (DHB11_HUMAN)	6.8
1zs6	Nucleoside diphosphate kinase 3(NDK3_HUMAN)	6.8
2dyl	Dual specificity mitogen-activated protein kinase kinase 7(MP2K7_HUMAN)	6.8
2i0e	Protein kinase C beta type (KPCB_HUMAN)	6.8
2onb	Thymidylate synthase (TYSY_HUMAN)	6.8
2q8g	[Pyruvate dehydrogenase [lipoamide]] kinase isozyme 1, mitochondrial	6.8
2xhd	Glutamate receptor 2 (GRIA2_HUMAN)	6.8
1eba	Erythropoietin receptor (EPOR_HUMAN)	6.7
1k3a	Insulin receptor substrate 1 (IRS1_HUMAN)	6.7
1rs0	Complement factor B (CFAB_HUMAN)	6.7
1t31	Chymase (CMA1_HUMAN)	6.7
1t5c	Centromeric protein E (CENPE_HUMAN)	6.7
1z6j	Coagulation factor VII Tissue factor(FA7_HUMAN/TF_HUMAN)	6.7
2hv8	Ras-related protein Rab-11A (RB11A_HUMAN)	6.7
2wex	Apolipoprotein M (APOM_HUMAN)	6.7
1j78	Vitamin D-binding protein (VTDB_HUMAN)	6.6
1mmr	Matrilysin (MMP7_HUMAN)	6.6
1mq0	Cytidine deaminase (CDD_HUMAN)	6.6
1t32	Cathepsin G (CATG_HUMAN)	6.6
2ewp	Estrogen-related receptor gamma (ERR3_HUMAN)	6.6
2jaj	N(G),N(G)-dimethylarginine dimethylaminohydrolase 1 (DDAH1_HUMAN)	6.6
2on3	Ornithine decarboxylase (DCOR_HUMAN)	6.6
1d5z	HLA class II histocompatibility antigen, DRB1-4 beta chain (2B14_HUMAN)	6.5
1wda	Protein-arginine deiminase type-4 (PADI4_HUMAN)	6.5
1x70	Dipeptidyl peptidase 4 (DPP4_HUMAN)	6.5
2hw6	MAP kinase-interacting serine/threonine-protein kinase 1(MKNK1_HUMAN)	6.5
2oju	Peptidyl-prolyl cis-trans isomerase-like 3 (PPIL3_HUMAN)	6.5

2z7x	Toll-like receptor 1 Toll-like receptor 2(TLR1_HUMAN/TLR2_HUMAN)	6.5
1i0e	Creatine kinase M-type(KCRM_HUMAN)	6.4
1kpe	Histidine triad nucleotide-binding protein 1 (HINT1_HUMAN)	6.4
1nuh	Glucose-6-phosphate isomerase (G6PI_HUMAN)	6.4
1zcm	Calpain-1 catalytic subunit (CAN1_HUMAN)	6.4
2ofv	Proto-oncogene tyrosine-protein kinase LCK (LCK_HUMAN)	6.4
2q8i	[Pyruvate dehydrogenase [lipoamide]] kinase isozyme 3, mitochondrial	6.4
2rew	Peroxisome proliferator-activated receptor alpha (PPARA_HUMAN)	6.4
2v62	Serine/threonine-protein kinase VRK2(VRK2_HUMAN)	6.4
2v77	Carboxypeptidase A1 (CBPA1_HUMAN)	6.4
1bnq	Carbonic anhydrase 2 (CAH2_HUMAN)	6.3
1cf0	Profilin-1 (PROF1_HUMAN)	6.3
1qha	Hexokinase-1(HXK1_HUMAN)	6.3
1xd3	Ubiquitin carboxyl-terminal hydrolase isozyme L3 (UCHL3_HUMAN)	6.3
2ipk	HLA class II histocompatibility antigen, DRB1-1 beta chain (2B11_HUMAN)	6.3
2nzt	Hexokinase-2(HXK2_HUMAN)	6.3
2uzp	Protein kinase C gamma type (KPCG_HUMAN)	6.3
2vpg	B-cell CLL/lymphoma 9 protein Pygopus homolog 1	6.3
2wal	Growth arrest and DNA-damage-inducible protein GADD45 gamma	6.3
1qgi	Chitosanase (CHIS_BACCI)	6.2
1w6f	Arylamine N-acetyltransferase (NAT_MYCSM)	6.2
2jii	Serine/threonine-protein kinase VRK3(VRK3_HUMAN)	6.2
2nnq	Fatty acid-binding protein, adipocyte (FABP4_HUMAN)	6.2
2nwg	Stromal cell-derived factor 1 (SDF1_HUMAN)	6.2
2vwi	Serine/threonine-protein kinase OSR1(OXSR1_HUMAN)	6.2
1e2s	Arylsulfatase A (ARSA_HUMAN)	6.1
1osh	Bile acid receptor (NR1H4_HUMAN)	6.1
1r55	ADAM 33 (ADA33_HUMAN)	6.1
2c47	Casein kinase I isoform gamma-2 (KC1G2_HUMAN)	6.1
2vgb	Pyruvate kinase isozymes R/L(KPYR_HUMAN)	6.1
2wo1	Ephrin type-A receptor 4 (EPHA4_HUMAN)	6.1
1d1q	Low molecular weight phosphotyrosine protein phosphatase (PPAL_YEAST)	6
2odb	Cell division control protein 42 homolog@Serine/threonine-protein kinase PAK 6	6
1jr1	Inosine-5'-monophosphate dehydrogenase 2 (IMDH2_CRIGR)	5.9
2f0z	Sialidase-2 (NEUR2_HUMAN)	5.9
2hy3	Receptor-type tyrosine-protein phosphatase gamma (PTPRG_HUMAN)	5.9
2obf	Phenylethanolamine N-methyltransferase (PNMT_HUMAN)	5.9
2wqn	Serine/threonine-protein kinase Nek7(NEK7_HUMAN)	5.9
1cza	Hexokinase-1(HXK1_HUMAN)	5.8
1efn	Proto-oncogene tyrosine-protein kinase Fyn (FYN_HUMAN)	5.8
1hkn	Heparin-binding growth factor 1 (FGF1_HUMAN)	5.8
1j86	High affinity immunoglobulin epsilon receptor subunit alpha (FCERA_HU-MAN)	5.8
1s1s	Cell division protein zipA (ZIPA_ECOLI)	5.8
1sr5	Antithrombin-III (ANT3_HUMAN)	5.8
1tev	UMP-CMP kinase(KCY_HUMAN)	5.8
1u4l	C-C motif chemokine 5 (CCL5_HUMAN)	5.8
2ggu	Pulmonary surfactant-associated protein D (SFTPD_HUMAN)	5.8

2xfo	Amine oxidase [flavin-containing] B (AOFB_HUMAN)	5.8
1mg4	Serine/threonine-protein kinase DCLK1(DCLK1_HUMAN)	5.7
2aeb	Arginase-1 (ARGI1_HUMAN)	5.7
2wor	Protein S100-A7 (S10A7_HUMAN)	5.7
1w80	Synaptojanin-1 (SYNJ1_HUMAN)	5.6
1xr9	HLA class I histocompatibility antigen, B-15 alpha chain (1B15_HUMAN)	5.6
2i3i	Baculoviral IAP repeat-containing protein 7 (BIRC7_HUMAN)	5.6
1dfp	Complement factor D (CFAD_HUMAN)	5.5
1t83	Low affinity immunoglobulin gamma Fc region receptor III-B (FCG3B_HU-MAN)	5.4
2qou	30S ribosomal protein S9 (RS9_ECOLI)	5.4
2zks	Granzyme M (GRAM_HUMAN)	5.4
1g1s	P-selectin (LYAM3_HUMAN)	5.3
1l5g	Integrin alpha-V (ITAV_HUMAN)	5.3
2bo9	Carboxypeptidase A4 Latexin(CBPA4_HUMAN/LXN_HUMAN)	5.3
2r2n	Kynurenine/alpha-aminoadipate aminotransferase mitochondrial (AADAT_HUMAN)	5.3
1auq	von Willebrand factor (VWF_HUMAN)	5.2
1dx4	Acetylcholinesterase (ACES_DROME)	5.1
1elv	Complement C1s subcomponent (C1S_HUMAN)	5.1
1kjr	Galectin-3 (LEG3_HUMAN)	5.1
2hxs	Ras-related protein Rab-28 (RAB28_HUMAN)	5.1
2prh	Dihydroorotate dehydrogenase, mitochondrial (PYRD_HUMAN)	5.1
2uz9	Guanine deaminase (GUAD_HUMAN)	5.1
1aii	Annexin A3 (ANXA3_HUMAN)	5
1bm7	Transthyretin (TTHY_HUMAN)	5
1czs	Coagulation factor V (FA5_HUMAN)	5
1g86	Eosinophil lysophospholipase (LPPL_HUMAN)	5
1l9n	Protein-glutamine gamma-glutamyltransferase E (TGM3_HUMAN)	5
2f8z	Farnesyl pyrophosphate synthetase (FPPS_HUMAN)	5
2wr6	Retinol-binding protein 4 (RET4_HUMAN)	4.9
2x8b	Acetylcholinesterase (ACES_HUMAN)	4.9
2xwc	Tumor protein p73 (P73_HUMAN)	4.9
1h0c	Serine--pyruvate aminotransferase (SPYA_HUMAN)	4.8
2huw	Growth factor receptor-bound protein 2 (GRB2_HUMAN)	4.8
1ft4	Tumor necrosis factor receptor superfamily member 1A (TNR1A_HUMAN)	4.7
1nrp	Proteinase-activated receptor 1 (PAR1_HUMAN)	4.7
2har	Vitamin D3 receptor (VDR_HUMAN)	4.7
1n7d	Low-density lipoprotein receptor (LDLR_HUMAN)	4.6
1pq6	Oxysterols receptor LXR-beta (NR1H2_HUMAN)	4.6
2v8e	Complement factor H (CFAH_HUMAN)	4.6
1nbf	Ubiquitin carboxyl-terminal hydrolase 7 (UBP7_HUMAN)	4.3
2r4b	Receptor tyrosine-protein kinase erbB-4 (ERBB4_HUMAN)	4.3
1au1	Interferon beta (IFNB_HUMAN)	4.2
1rqd	Deoxyhypusine synthase (DHYS_HUMAN)	4.2
1lgp	E3 ubiquitin-protein ligase CHFR (CHFR_HUMAN)	4.1
2dyb	Neutrophil cytosol factor 4 (NCF4_HUMAN)	4.1
2qkh	Gastric inhibitory polypeptide receptor Gastric inhibitory polypeptide)	4.1
1w70	Neutrophil cytosol factor 1 (NCF1_HUMAN)	4

1k5r	65 kDa Yes-associated protein (YAP1_HUMAN)	3.9
1f9p	Platelet basic protein (CXCL7_HUMAN)	3.7
1lhv	Sex hormone-binding globulin (SHBG_HUMAN)	3.7
2ayo	Ubiquitin carboxyl-terminal hydrolase 14 (UBP14_HUMAN)	3.7
2ra4	C-C motif chemokine 13 (CCL13_HUMAN)	3.7
2e1q	Xanthine dehydrogenase/oxidase (XDH_HUMAN)	3.3
2hrq	Liver carboxylesterase 1 (EST1_HUMAN)	3.3
2ibi	Ubiquitin carboxyl-terminal hydrolase 2 (UBP2_HUMAN)	3.3
1fga	Heparin-binding growth factor 2 (FGF2_HUMAN)	3.2
1hy3	Estrogen sulfotransferase (ST1E1_HUMAN)	3.2
2a3r	Sulfotransferase 1A3/1A4 (ST1A3_HUMAN)	2.6
1sr7	Progesterone receptor (PRGR_HUMAN)	2.3
1xap	Retinoic acid receptor beta (RARB_HUMAN)	1.2
1uou	Thymidine phosphorylase (TYPH_HUMAN)	0.7
2pnu	Androgen receptor (ANDR_HUMAN)	0.7
2wnv	Complement C1q subcomponent subunit A B C	0.6
2a3i	Mineralocorticoid receptor (MCR_HUMAN)	-0.6
1u3u	Alcohol dehydrogenase 1B (ADH1B_HUMAN)	-0.9
1w6k	Lanosterol synthase (ERG7_HUMAN)	-1.2
2qcg	Uridine 5'-monophosphate synthase (PYR5_HUMAN)	-1.6
1u3t	Alcohol dehydrogenase 1A (ADH1A_HUMAN)	-2.2
2pqt	Arylamine N-acetyltransferase 1 (ARY1_HUMAN)	-2.4
1lyb	Cathepsin D (CATD_HUMAN)	-2.5
1xvp	Nuclear receptor subfamily 1 group I member 3 (NR1I3_HUMAN)	-2.7
1a7c	Plasminogen activator inhibitor 1 (PAI1_HUMAN)	-3.6
2gwh	Sulfotransferase 1C4 (ST1C4_HUMAN)	-3.8
1exa	Retinoic acid receptor gamma (RARG_HUMAN)	-4
2znt	Glutamate receptor, ionotropic kainate 1 (GRIK1_HUMAN)	-4.1
2qro	Deoxycytidine kinase (DCK_HUMAN)	-4.8
1ls6	Sulfotransferase 1A1 (ST1A1_HUMAN)	-6
1w4n	Phenylethylamine oxidase (PAOX_ARTGO)	-10.8
2q3z	Protein-glutamine gamma-glutamyltransferase 2 (TGM2_HUMAN)	-10.8
1.00E+51	Delta-aminolevulinic acid dehydratase (HEM2_HUMAN)	-10.9
1p49	Steryl-sulfatase (STS_HUMAN)	-12
2p8u	Hydroxymethylglutaryl-CoA synthase, cytoplasmic (HMCS1_HUMAN)	-13
1fsu	Arylsulfatase B (ARSB_HUMAN)	-17.6
2c11	Membrane primary amine oxidase (AOC3_HUMAN)	-18.3
1m5b	Glutamate receptor 2 (GRIA2_RAT)	-19.8
1ym9	M-phase inducer phosphatase 2 (MPIP2_HUMAN)	-24.5
2hhj	Bisphosphoglycerate mutase (PMGE_HUMAN)	-25.2
2hei	Ras-related protein Rab-5B (RAB5B_HUMAN)	-42
2pfg	Carbonyl reductase [NADPH] 1 (CBR1_HUMAN)	-43.4
1evu	Coagulation factor XIII A chain (F13A_HUMAN)	-46
1stf	Cystatin-B (CYTB_HUMAN)	-56.8

Table S.1. Docking results of Tunicamycin with protein targets. The spatial data file (.sdf) for tunicamycin C were subjected to LigPrep ligand energy minimization in an implicit solvent and the resulting energy-minimised ligand was used as a query on the HighThoughput Docking website. The algorithm elucidated the most likely targets to interact with tunicamycin C. A high docking score suggested a strong ligand and protein interaction.

Table S2: KEGG Pathway analysis for Targeted proteins for Tunicamycin C

Term	Overlap	P-value	Adjusted P-value	Odds Ratio	Combined Score
Neuroactive ligand-receptor interaction	17/341	5.37E-13	1.12E-10	13.00438	367.3997
Starch and sucrose metabolism	13/363	5.67E-12	5.90E-10	64.53247	1671.134
Vascular smooth muscle contraction	11/133	4.04E-11	2.80E-09	20.9838	502.2142
Carbohydrate digestion and absorption	17/380	5.64E-11	2.93E-09	46.30536	1092.717
Ras signaling pathway	12/232	1.18E-09	4.92E-08	12.78182	262.745
HIF-1 signaling pathway	9/109	2.65E-09	9.19E-08	20.4869	404.589
Proteoglycans in cancer	11/205	4.13E-09	1.23E-07	13.14797	253.8105
Galactose metabolism	11/475	6.98E-09	1.81E-07	53.01067	995.5833
Pyrimidine metabolism	20/637	9.00E-09	2.08E-07	31.86998	590.43
AGE-RAGE signaling pathway in diabetes	8/100	2.71E-08	5.64E-07	19.57708	341.0823
Pathways in cancer	15/531	3.41E-08	6.45E-07	6.958082	119.6296
Growth hormone synthesis, secretion and action	8/119	1.06E-07	1.70E-06	16.21048	260.3153
Sphingolipid signaling pathway	8/119	1.06E-07	1.70E-06	16.21048	260.3153
Pancreatic secretion	7/102	5.99E-07	8.90E-06	16.40012	234.9819
Insulin resistance	7/108	8.83E-07	1.23E-05	15.42118	214.9617
PI3K-Akt signaling pathway	11/354	1.05E-06	1.37E-05	7.380244	101.5767
Calcium signaling pathway	9/240	2.32E-06	2.84E-05	8.810121	114.2841
Relaxin signaling pathway	7/129	2.92E-06	3.37E-05	12.75318	162.5328
Estrogen signaling pathway	7/137	4.35E-06	4.53E-05	11.96353	147.6852
Insulin signaling pathway	7/137	4.35E-06	4.53E-05	11.96353	147.6852
Diabetic cardiomyopathy	8/203	6.06E-06	6.00E-05	9.188345	110.3883
Fc gamma R-mediated phagocytosis	35/582	7.26E-06	6.73E-05	14.51502	171.7602
Inflammatory regulation of TRP channels	35/947	7.70E-06	6.73E-05	14.35652	169.0329
Rap1 signaling pathway	8/210	7.77E-06	6.73E-05	8.866787	104.3214
Lipid and atherosclerosis	8/215	9.23E-06	7.63E-05	8.650417	100.2892
cAMP signaling pathway	8/216	9.54E-06	7.63E-05	8.608392	99.51074
Lysine degradation	23/132	1.29E-05	9.97E-05	18.80068	211.6115
Drug metabolism	6/108	1.35E-05	1.00E-04	12.94248	145.1469
Chemical carcinogenesis	8/239	1.98E-05	1.42E-04	7.742227	83.82888
Shigellosis	8/246	2.44E-05	1.69E-04	7.511841	79.77968
Bladder cancer	15/67	4.40E-05	2.96E-04	23.34548	234.1596
Fluid shear stress and atherosclerosis	6/139	5.61E-05	3.65E-04	9.910276	97.00186
Type II diabetes mellitus	16/893	6.97E-05	4.39E-04	20.56108	196.7993
GnRH signaling pathway	34/90	8.54E-05	4.96E-04	12.37263	115.9099
Salivary secretion	34/90	8.54E-05	4.96E-04	12.37263	115.9099
MAPK signaling pathway	8/294	8.58E-05	4.96E-04	6.235855	58.38813
Prostate cancer	35/551	1.04E-04	5.86E-04	11.8323	108.4811

MicroRNAs in cancer	8/310	1.24E-04	6.78E-04	5.900662	53.08758
Hepatitis B	6/162	1.31E-04	6.88E-04	8.439316	75.46376
Longevity regulating pathway	5/102	1.32E-04	6.88E-04	11.21955	100.197
Parathyroid hormone synthesis, secretion, action	5/106	1.59E-04	8.04E-04	10.77304	94.25888
VEGF signaling pathway	21/641	1.85E-04	9.18E-04	15.69091	134.8386
Cholinergic synapse	5/113	2.14E-04	0.001032	10.07123	85.10348
Leukocyte transendothelial migration	5/114	2.23E-04	0.001032	9.978324	83.90854
Neomycin, kanamycin, gentamicin biosynthesis	44/597	2.26E-04	0.001032	141.1418	1184.965
alpha-Linolenic acid metabolism	44/645	2.28E-04	0.001032	29.15249	244.44
Linoleic acid metabolism	44/649	3.58E-04	0.001554	24.66253	195.7187
Central carbon metabolism in cancer	25/659	3.59E-04	0.001554	13.06851	103.6761
Focal adhesion	6/201	4.18E-04	0.001776	6.73812	52.4154
Gastric acid secretion	27/851	4.91E-04	0.002042	11.97585	91.24728
Autophagy	5/137	5.20E-04	0.002121	8.230103	62.23363
Human cytomegalovirus infection	6/225	7.57E-04	0.002986	5.99239	43.06116
Gastric cancer	5/149	7.61E-04	0.002986	7.539683	54.14253
Morphine addiction	33/329	9.68E-04	0.003729	9.903548	68.73231
Small cell lung cancer	33/695	0.001008	0.003814	9.790514	67.54835
Fat digestion and absorption	15/766	0.001151	0.004274	16.01935	108.4088
cGMP-PKG signaling pathway	5/167	0.001267	0.004503	6.695835	44.66803
Aldosterone synthesis and secretion	35/886	0.001275	0.004503	9.162812	61.06504
Salmonella infection	6/249	0.001277	0.004503	5.393964	35.94065
Other types of O-glycan biosynthesis	17/227	0.001491	0.005169	14.56012	94.76035
Ether lipid metabolism	17/958	0.001683	0.005737	13.92567	88.9491
Glucagon signaling pathway	4/107	0.001763	0.005889	8.358379	52.99649
Cysteine and methionine metabolism	18/323	0.001784	0.005889	13.62869	86.25608
Endocrine-regulated calcium reabsorption	19/419	0.00211	0.006857	12.80903	78.91737
Chemokine signaling pathway	5/192	0.002336	0.007396	5.793324	35.10362
Regulation of lipolysis in adipocytes	20/149	0.002347	0.007396	12.31514	74.56497
Thyroid hormone signaling pathway	4/121	0.002759	0.008564	7.353029	43.33133
Platelet activation	4/124	0.003013	0.009217	7.168116	41.60881
Arachidonic acid metabolism	22/341	0.003153	0.009505	11.03782	63.57044
Purine metabolism	4/129	0.003473	0.010319	6.879652	38.95834
Human immunodeficiency virus 1 infection	5/212	0.003575	0.010437	5.228274	29.45467
GnRH secretion	23/437	0.003613	0.010437	10.49339	59.00739
Dopaminergic synapse	4/132	0.00377	0.010742	6.717391	37.4877
Glycolysis / Gluconeogenesis	24/532	0.004111	0.011402	10	54.9406
Long-term potentiation	24/532	0.004111	0.011402	10	54.9406
Amphetamine addiction	25/263	0.004465	0.012063	9.695992	52.46875

Renin secretion	25/263	0.004465	0.012063	9.695992	52.46875
Measles	4/139	0.00453	0.01208	6.366828	34.36223
Melanoma	26/359	0.005031	0.01308	9.273025	49.07462
Non-small cell lung cancer	26/359	0.005031	0.01308	9.273025	49.07462
p53 signaling pathway	26/724	0.005228	0.013426	9.140092	48.01905
Glioma	27/454	0.005637	0.014127	8.885305	46.01125
Thyroid hormone synthesis	27/454	0.005637	0.014127	8.885305	46.01125
Pancreatic cancer	27/820	0.005849	0.014483	8.763146	45.05592
Adrenergic signaling in cardiomyocytes	4/150	0.005922	0.014492	5.883859	30.17873
Oxytocin signaling pathway	4/154	0.006492	0.01552	5.725797	28.84213
mTOR signaling pathway	4/154	0.006492	0.01552	5.725797	28.84213
JAK-STAT signaling pathway	4/162	0.007738	0.018289	5.433682	26.41657
ErbB signaling pathway	31/107	0.007967	0.018619	7.797797	37.68251
Insulin secretion	31/472	0.008227	0.018804	7.703459	36.97957
Taste transduction	31/472	0.008227	0.018804	7.703459	36.97957
Wnt signaling pathway	4/166	0.008416	0.019028	5.298443	25.31394
Gap junction	32/203	0.008761	0.019404	7.521442	35.63267
Hepatocellular carcinoma	4/168	0.008769	0.019404	5.233298	24.78753
GABAergic synapse	32/568	0.009035	0.019782	7.433608	34.98719
IL-17 signaling pathway	34/394	0.010483	0.022714	7.023396	32.01249
Fructose and mannose metabolism	12/086	0.010928	0.023434	13.63967	61.60202
NOD-like receptor signaling pathway	4/181	0.011303	0.023979	4.845738	21.7219
Circadian entrainment	35/490	0.011413	0.023979	6.798216	30.40849
Choline metabolism in cancer	35/855	0.011733	0.024163	6.726316	29.9008
Glycerophospholipid metabolism	35/855	0.011733	0.024163	6.726316	29.9008
Progesterone-mediated oocyte maturation	3/100	0.012389	0.025263	6.586964	28.92314
Melanogenesis	3/101	0.012724	0.025696	6.519421	28.45231
Amoebiasis	3/102	0.013065	0.0259	6.453242	27.99294
Neutrophil extracellular trap formation	4/189	0.013075	0.0259	4.634313	20.09941
African trypanosomiasis	13/547	0.013615	0.0263	12.07842	51.89612
Aldosterone-regulated sodium reabsorption	13/547	0.013615	0.0263	12.07842	51.89612
NF-kappa B signaling pathway	3/104	0.013762	0.0263	6.324816	27.10706
Transcriptional misregulation in cancer	4/192	0.013782	0.0263	4.559667	19.5354
Primary immunodeficiency	13/912	0.014327	0.02709	11.74232	49.85354
Toxoplasmosis	3/112	0.01676	0.031406	5.858242	23.95295
Serotonergic synapse	3/113	0.017158	0.031866	5.804692	23.59764
Glutamatergic synapse	3/114	0.017562	0.032327	5.752107	23.25011
Neurotrophin signaling pathway	3/119	0.01966	0.03587	5.502781	21.62143
Human T-cell leukemia virus 1 infection	4/219	0.021255	0.038443	3.981598	15.33385

Amino sugar and nucleotide sugar metabolism	17/564	0.022288	0.039965	9.185014	34.93712
Arginine and proline metabolism	18/295	0.024056	0.042047	8.801418	32.80628
N-Glycan biosynthesis	18/295	0.024056	0.042047	8.801418	32.80628
Vibrio cholerae infection	18/295	0.024056	0.042047	8.801418	32.80628
FoxO signaling pathway	3/131	0.025236	0.043742	4.983871	18.33811
Apelin signaling pathway	3/137	0.028311	0.048666	4.759268	16.9645
Hedgehog signaling pathway	20/486	0.029688	0.050615	7.821119	27.5071
Apoptosis	3/142	0.031018	0.052454	4.586911	15.93114
Spinocerebellar ataxia	3/143	0.031576	0.052966	4.553917	15.73544
Long-term depression	21/947	0.033704	0.055892	7.280264	24.68105
Breast cancer	3/147	0.033858	0.055892	4.426523	14.98637
Cell adhesion molecules	3/148	0.034441	0.055967	4.395773	14.80713
Retrograde endocannabinoid signaling	3/148	0.034441	0.055967	4.395773	14.80713
Necroptosis	3/159	0.041204	0.065931	4.08354	13.02331
Acute myeloid leukemia	24/504	0.041207	0.065931	6.493944	20.71014
Fc epsilon RI signaling pathway	24/869	0.042326	0.067204	6.395229	20.224
Renal cell carcinoma	25/235	0.043456	0.068476	6.29946	19.75514
Tight junction	3/169	0.047889	0.074895	3.8356	11.65586
Influenza A	3/172	0.049993	0.077601	3.76694	11.28527
Chronic myeloid leukemia	27/791	0.051674	0.079616	5.701553	16.89258
B cell receptor signaling pathway	29/618	0.057856	0.088485	5.339348	15.2161
Alcoholism	3/186	0.060394	0.091692	3.476291	9.757505
CRC	31/444	0.06428	0.096886	5.020263	13.77814
Kaposi sarcoma-associated herpesvirus infection	3/193	0.065945	0.09868	3.347029	9.100346
Bile secretion	32/905	0.069584	0.103382	4.791103	12.76932
Epstein-Barr virus infection	3/202	0.073415	0.1083	3.194197	8.342066
Human papillomavirus infection	4/331	0.075199	0.110151	2.602978	6.73551
Phosphatidylinositol signaling system	35/462	0.079196	0.115194	4.436506	11.25024
Glycosaminoglycan degradation	44/580	0.087403	0.125626	11.62924	28.34313
Regulation of actin cytoskeleton	3/218	0.087576	0.125626	2.954089	7.193941
C-type lectin receptor signaling pathway	2/104	0.089194	0.126206	4.13058	9.983378
T cell receptor signaling pathway	2/104	0.089194	0.126206	4.13058	9.983378
One carbon pool by folate	44/581	0.091787	0.128133	11.01662	26.31079
Steroid biosynthesis	44/581	0.091787	0.128133	11.01662	26.31079
Terpenoid backbone biosynthesis	44/583	0.100494	0.138277	9.966416	22.8994
Coronavirus disease	3/232	0.100839	0.138277	2.771517	6.358498
TNF signaling pathway	2/112	0.101048	0.138277	3.828627	8.775815
Mannose type O-glycan biosynthesis	44/584	0.104816	0.14157	9.512919	21.45681
Renin-angiotensin system	44/584	0.104816	0.14157	9.512919	21.45681

Parkinson disease	3/249	0.117951	0.158283	2.57776	5.509928
Oocyte meiosis	2/129	0.127537	0.17005	3.313285	6.823198
Natural killer cell mediated cytotoxicity	2/131	0.130755	0.173229	3.261587	6.635479
Yersinia infection	2/137	0.140517	0.184984	3.115682	6.114306
Stem cell pluripotency	2/143	0.150431	0.19679	2.982194	5.649021
Nicotinate and nicotinamide metabolism	12/785	0.155109	0.201642	6.151703	11.46448
Phospholipase D signaling pathway	2/148	0.158798	0.204368	2.879335	5.29833
DNA replication	13/150	0.159172	0.204368	5.975639	10.98186
Cushing syndrome	2/155	0.170653	0.217187	2.746628	4.856371
Various types of N-glycan biosynthesis	14/246	0.171244	0.217187	5.503047	9.711048
Cellular senescence	2/156	0.172359	0.217277	2.728654	4.797451
Vasopressin-regulated water reabsorption	16/72	0.190984	0.239305	4.861934	8.049253
Pathways of neurodegeneration	4/475	0.194464	0.241275	1.793871	2.93748
Glycosphingolipid biosynthesis	16/438	0.194876	0.241275	4.751196	7.770074
Protein processing in endoplasmic reticulum	2/171	0.198257	0.244008	2.484578	4.020524
Cocaine addiction	17/899	0.210259	0.257258	4.354386	6.790303
Tuberculosis	2/180	0.214018	0.258862	2.357877	3.635124
Malaria	18/264	0.214059	0.258862	4.265306	6.57499
Axon guidance	2/182	0.217538	0.260407	2.331442	3.556338
Ovarian steroidogenesis	18/629	0.217841	0.260407	4.179789	6.369963
Glycosaminoglycan biosynthesis	19/360	0.225351	0.267845	4.018623	5.988141
Glutathione metabolism	20/821	0.240157	0.283822	3.730827	5.321881
Endometrial cancer	21/186	0.243815	0.285215	3.665189	5.17285
Pathogenic Escherichia coli infection	2/197	0.244078	0.285215	2.150464	3.032731
Viral carcinogenesis	2/203	0.25474	0.296011	2.085636	2.852133
Cortisol synthesis and secretion	23/743	0.268935	0.31077	3.263158	4.285455
Mitophagy	24/838	0.279447	0.321132	3.116575	3.973459
Adipocytokine signaling pathway	25/204	0.282917	0.323334	3.070588	3.876929
Prolactin signaling pathway	25/569	0.286371	0.325493	3.025934	3.783832
Adherens junction	25/934	0.289808	0.32761	2.982556	3.694001
Leishmaniasis	28/126	0.310092	0.348644	2.74626	3.215561
Antigen processing and presentation	28/491	0.313416	0.350487	2.710458	3.144738
PD-L1 and PD-1 checkpoint pathway in cancer	32/509	0.348954	0.388141	2.370335	2.495525
Rheumatoid arthritis	33/97	0.36142	0.399869	2.266819	2.306973
Staphylococcus aureus infection	34/70	0.367565	0.404516	2.218365	2.220264
Dilated cardiomyopathy	35/65	0.370615	0.405726	2.194903	2.178643
Prion disease	2/273	0.377862	0.411318	1.541415	1.500145
Hematopoietic cell lineage	36/161	0.379678	0.411318	2.12739	2.060231
Chagas disease	1/102	0.388612	0.418816	2.063887	1.95073

Toll-like receptor signaling pathway	1/104	0.394498	0.422967	2.023608	1.882242
Th17 cell differentiation	1/107	0.403221	0.430102	1.966038	1.785695
Herpes simplex virus 1 infection	3/498	0.429297	0.45558	1.264842	1.069559
AMPK signaling pathway	1/120	0.439606	0.464152	1.750111	1.438376
Cell cycle	1/124	0.450353	0.473098	1.692854	1.35043
Osteoclast differentiation	1/127	0.458279	0.479005	1.652297	1.28925
Lysosome	1/128	0.460896	0.479332	1.639204	1.2697
Amyotrophic lateral sclerosis	2/364	0.523898	0.541999	1.148584	0.742512
Non-alcoholic fatty liver disease	1/155	0.527011	0.541999	1.349966	0.864699
Alzheimer disease	2/369	0.531254	0.541999	1.132645	0.716416
Hepatitis C	1/157	0.531576	0.541999	1.332524	0.842034
Hippo signaling pathway	1/163	0.545012	0.552987	1.282781	0.778581
RNA transport	1/186	0.593073	0.598831	1.121991	0.58617
Thermogenesis	1/232	0.67463	0.677889	0.896468	0.352842
Olfactory transduction	1/440	0.882429	0.882429	0.466731	0.058377

Table S2: KEGG pathway analysis for Targeted proteins for Tunicamycin C. To establish the mechanisms of tunicamycin C on pathways involved in colorectal cancer and glycosylation, the 93 protein targets were subjected to KEGG pathway analysis using the STRING database. A total of 108 enriched KEGG pathways met the FDR screening threshold ≤ 0.05. Pathways of interest are highlighted in yellow.

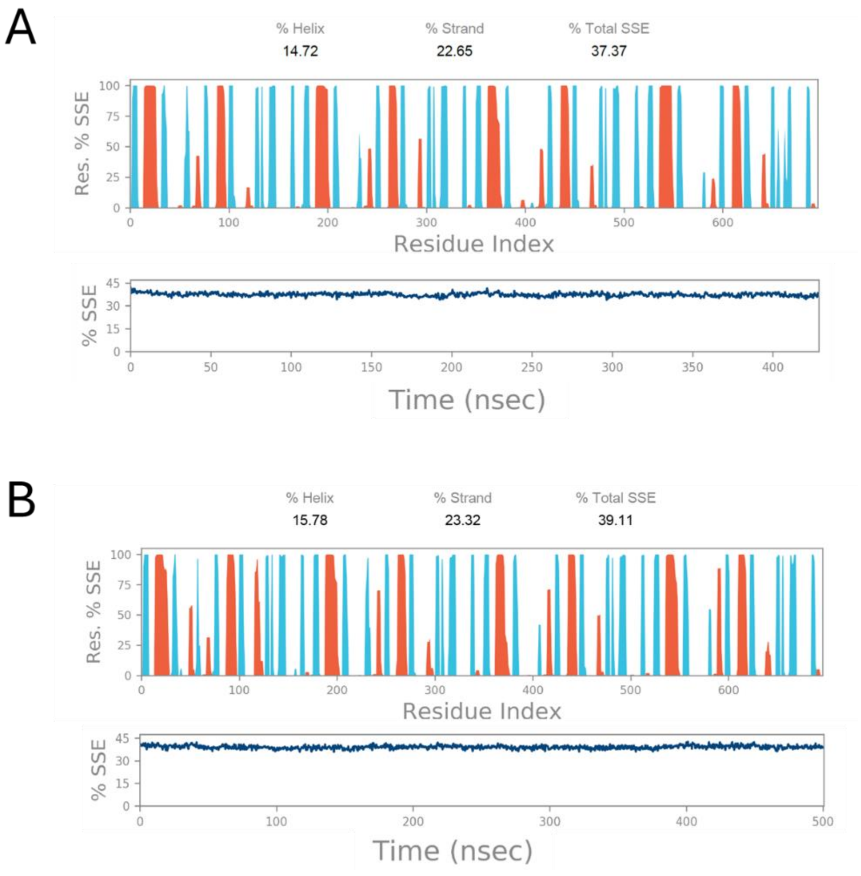

Figure S1: Protein secondary structure elements (SSE) of the Apo-TK1 (A) and Tunicamycin-bound-TK1 (B). This was monitored throughout the simulation showing the SSE distribution by residue index throughout the protein structure (top panel) and summary of the SSE composition for each trajectory frame over the course of the simulation (bottom panel).
